# Can we reduce the workload of mammographic screening by automatic identification of normal exams with artificial intelligence? A feasibility study

**DOI:** 10.1007/s00330-019-06186-9

**Published:** 2019-04-16

**Authors:** Alejandro Rodriguez-Ruiz, Kristina Lång, Albert Gubern-Merida, Jonas Teuwen, Mireille Broeders, Gisella Gennaro, Paola Clauser, Thomas H. Helbich, Margarita Chevalier, Thomas Mertelmeier, Matthew G. Wallis, Ingvar Andersson, Sophia Zackrisson, Ioannis Sechopoulos, Ritse M. Mann

**Affiliations:** 1 Department of Radiology and Nuclear Medicine, Radboud University Medical Center, PO Box 9101, 6500 HB Nijmegen, The Netherlands; 2 ScreenPoint Medical BV, Stationplein 26, 6512 AB Nijmegen, The Netherlands; 3 Institute for Biomedical Engineering, ETH Zurich, Gloriastrasse 35, 8092 Zürich, Switzerland; 4 Department for Health Evidence, Radboud University Medical Center, P.O. Box 9101, 6500 HB Nijmegen, The Netherlands; 5 Dutch Expert Centre for Screening (LRCB), Wijchenseweg 101, 6538 SW Nijmegen, The Netherlands; 6 Veneto Institute of Oncology (IOV)–IRCCS, via Gattamelata 64, 35128 Padua, Italy; 7 Department of Biomedical Imaging and Image-Guided Therapy, Division of Molecular and Gender Imaging, Medical University of Vienna, Waehringer Guertel 18-20, 1090 Vienna, Austria; 8 Medical Physics Group, Radiology Department, Faculty of Medicine, Universidad Complutense de Madrid, Pza. Ramón y Cajal s/n, 28040 Madrid, Spain; 9 Siemens Healthcare GmbH, Diagnostic Imaging, X-Ray Products, Technology & Concepts, Siemensstr. 3, 91301 Forchheim, Germany; 10 Cambridge Breast Unit and NIHR Biomedical Research Unit, Box 97, Cambridge University Hospitals NHS Foundation Trust, Cambridge Biomedical Campus, Hills Road, Cambridge, CB2 0QQ UK; 11 Unilabs Breast Center, Skåne University Hospital, Jan Waldenströms gata 22, SE-20502 Malmö, Sweden; 12 Diagnostic Radiology, Department of Translational Medicine, Lund University, Skåne University Hospital, SE-20502 Malmö, Sweden

**Keywords:** Mammography, Breast cancer, Screening, Deep learning, Artificial intelligence

## Abstract

**Purpose:**

To study the feasibility of automatically identifying normal digital mammography (DM) exams with artificial intelligence (AI) to reduce the breast cancer screening reading workload.

**Methods and materials:**

A total of 2652 DM exams (653 cancer) and interpretations by 101 radiologists were gathered from nine previously performed multi-reader multi-case receiver operating characteristic (MRMC ROC) studies. An AI system was used to obtain a score between 1 and 10 for each exam, representing the likelihood of cancer present. Using all AI scores between 1 and 9 as possible thresholds, the exams were divided into groups of low- and high likelihood of cancer present. It was assumed that, under the pre-selection scenario, only the high-likelihood group would be read by radiologists, while all low-likelihood exams would be reported as normal. The area under the reader-averaged ROC curve (AUC) was calculated for the original evaluations and for the pre-selection scenarios and compared using a non-inferiority hypothesis.

**Results:**

Setting the low/high-likelihood threshold at an AI score of 5 (high likelihood > 5) results in a trade-off of approximately halving (− 47%) the workload to be read by radiologists while excluding 7% of true-positive exams. Using an AI score of 2 as threshold yields a workload reduction of 17% while only excluding 1% of true-positive exams. Pre-selection did not change the average AUC of radiologists (inferior 95% CI > − 0.05) for any threshold except at the extreme AI score of 9.

**Conclusion:**

It is possible to automatically pre-select exams using AI to significantly reduce the breast cancer screening reading workload.

**Key Points:**

*• There is potential to use artificial intelligence to automatically reduce the breast cancer screening reading workload by excluding exams with a low likelihood of cancer.*

*• The exclusion of exams with the lowest likelihood of cancer in screening might not change radiologists’ breast cancer detection performance.*

*• When excluding exams with the lowest likelihood of cancer, the decrease in true-positive recalls would be balanced by a simultaneous reduction in false-positive recalls.*

## Introduction

Population-based screening programs with digital mammography (DM) reduce mortality from breast cancer due to the earlier detection of the disease [1, 2], but their efficiency is continuously under discussion [3, 4]. False-positive findings [5] lead to negative effects such as unnecessary workup, participant anxiety and reluctance to re-attend screening, as well as a reduction in cost-effectiveness [6]. On the other hand, since the program-based sensitivity of screening is approximately 75% [7], false-negative findings may lead to false reassurance and ultimately a delayed cancer detection. One of the reasons why mammographically visible cancers are missed is the low prevalence of cancer (approximately, 10 per thousand) in a screening population [8, 9].

Computer-aided detection (CAD) systems to improve mammography reading have been used since the beginning of this century. However, so far no study has found any direct improvement in screening outcomes, likely because of the low specificity of these traditional CAD systems [10, 11]. The recent breakthrough in artificial intelligence (AI) performance, based on the use of deep learning algorithms, is now closing the gap between human and computer performance in many applications related to medical imaging [12]. Novel AI systems may, therefore, be able to improve the performance and efficiency of population-based screening programs [13]. For mammography evaluation, deep learning–based systems have demonstrated a stand-alone performance as good as radiologists [14], as well as a significant improvement of radiologists’ breast cancer detection accuracy when used for decision support [15]. However, this radiologist-like performance may enable other uses of AI for mammography evaluation in screening. Of particular interest are approaches aimed at reducing workload, considering the increasing scarcity of (breast) radiologists in some countries [16–18].

In this work, we explore the possibility of using an AI system to pre-select likely-normal mammograms. This was done using an AI system that provides an exam-based score denoting the likelihood of cancer present in the mammogram. We analyzed the effects on performance of excluding exams with a low score (i.e., low likelihood of cancer present) from human reading, which would reduce the screening workload for radiologists and increase the cancer prevalence in the actually evaluated images.

## Materials and methods

### Data and population characteristics

Digital mammograms were collected from nine previously performed multi-reader multi-case (MRMC) observer studies [19–26]. The review board at each institution waived local ethical approval and informed consent or approved the use of the anonymized patient data for retrospective research.

All the datasets of the MRMC studies were enriched with exams with cancer. The ground truth, in terms of cancer present, benign lesion present, or absence of abnormalities, of each DM exam, was confirmed by histopathology and/or at least 1 year of follow-up. During each MRMC study, each DM exam was evaluated by multiple breast radiologists who provided malignancy scores for each exam (BI-RADS and/or level of suspicion).

In total, 2654 exams (653 with cancer, 768 with benign lesions, 1233 normal) and readings by 101 radiologists (52% from the USA and 48% from Europe) were gathered (yielding 28,296 independent exam interpretations). Approximately, half the exams were from screening and half from clinical practice. Detailed information about the tumor histology was not available. The DM images were acquired with devices from four different vendors (Siemens Healthineers; Hologic Inc.; General Electric Healthcare; Sectra Mamea) across seven different countries. Further details of these nine studies have previously been reported elsewhere [14].

### Artificial intelligence system

For this study, we used an AI system dedicated to breast cancer detection in DM and digital breast tomosynthesis (Transpara 1.4.0, Screenpoint Medical BV). The system uses deep learning convolutional neural networks, feature classifiers, and image analysis algorithms to detect calcifications and soft tissue lesions in two different modules [14]. Based on these detected lesions and overall exam appearance, the AI system assigns an exam-based integer score denoting the likelihood that cancer is present in the exam (hereafter AI score, also known as Transpara Score). This AI score ranges between 1 and 10 (10 means high likelihood that a cancer is present in the exam). The AI score is calibrated so that approximately the same number of normal exams (10% of the total) is assigned to each AI score category. In a population with low prevalence of cancer (where most exams are normal), such as a screening population, it may therefore be expected that approximately 10% of the total exams are in each category. In a screening population, the 10% of exams scored 1 are predicted to have the lowest risk of harboring cancer (because category 1 has the lowest incidence of exams with cancer), while the 10% of exams scored 10 have the highest risk of harboring cancer (because category 10 contains the largest fraction of exams with cancer). Since the calibration of the AI score is performed only with screening mammograms without abnormalities, the AI score is independent to the composition of the datasets. However, the fact that all datasets used for this study were enriched with cancers implies that the found distribution of AI scores in our study is skewed towards higher numbers, since it should be expected that cancer cases are not evenly distributed over AI categories.

The AI system was trained, validated, and tested using an external database representative of screening containing over 9000 mammograms with cancer (one-third of which are presented as lesions with calcifications) and 180,000 mammograms without abnormalities. The AI score was also calibrated with this external database, using only the normal mammograms. The mammograms used in this study have never been used to train, validate, or test the algorithms. The mammograms originate from devices from four different vendors (Hologic; Siemens; General Electric; Philips) and institutions across Europe, USA, and Asia.

### Automated pre-selection of cases

For this study, the distribution of the normal exams and those containing benign or malignant lesions according to the ground truth was computed as a function of the AI score. To divide the exams into two groups (excluded and pre-selected for evaluation), we varied the threshold dividing these two groups across all possible AI scores, i.e., from 1 to 9. Consequently, the pre-selection scenarios included exams-to-be-evaluated as those with scores greater than 1, 2, 3, 4, 5, 6, 7, 8, or greater than 9 (equivalent to only pre-selected category 10). For each threshold, the characteristics of the exams in both groups were analyzed.

Under the pre-selection scenarios, we assumed that readers would only evaluate exams in the pre-selected group (high likelihood of cancer present), whereas exams in the low-likelihood group would automatically be assigned a “normal” classification. Workload reduction throughout the text is therefore expressed in terms of the number of exams that have to be read by the screening radiologists. Given the calibration of the AI system, a pre-selection threshold of 5, for instance, means that half of the exams in a screening program would be excluded from human reading. An estimation of how radiologists’ performance would change after pre-selection was calculated by a posteriori modification of the original radiologists’ scores: for the exams in the excluded group, all the radiologists’ scores were automatically modified to the lowest possible value (e.g., 1). This implies that we assumed invariance in human behavior for the pre-selected mammograms that were above the threshold and therefore should be evaluated.

### Statistics

The breast cancer detection accuracy of radiologists in the original scenario was compared with the simulated pre-selection scenario with a non-inferiority null hypothesis [27–31] based on the differences in the average area under the receiver operating characteristic curve (AUC). The non-inferiority margin was set at 0.05 in this study. Non-inferiority was concluded when the AUC difference “pre-selection scenario” – “original reading” was greater than 0 and the lower limit of the 95% confidence interval (CI) of the difference was greater than the non-inferiority margin. Confidence intervals were Bonferroni-corrected for multiple comparisons.

To obtain the average AUC across all our data, we used the public-domain iMRMC software for analysis (version 4.0.0, Division of Imaging, Diagnostics, and Software Reliability, OSEL/CDRH/FDA, Silver Spring, MD) [29, 30], which can handle not fully crossed study designs, such as the split-plot design resulting when pooling the nine datasets from this study [32, 33]. The reader-averaged ROC curves were created by averaging the reader-specific non-parametric (trapezoidal) curves along lines perpendicular to the chance line [34]. This average is area-preserving; its AUC is equal to the reader-averaged non-parametric AUCs. The analysis was not done per dataset, given the homogeneous performance of the AI system across datasets seen in Rodriguez-Ruiz et al [14]. We therefore assumed that no differences per dataset would be present in this study.

## Results

### Performance of the AI system

**Fig. 1:**
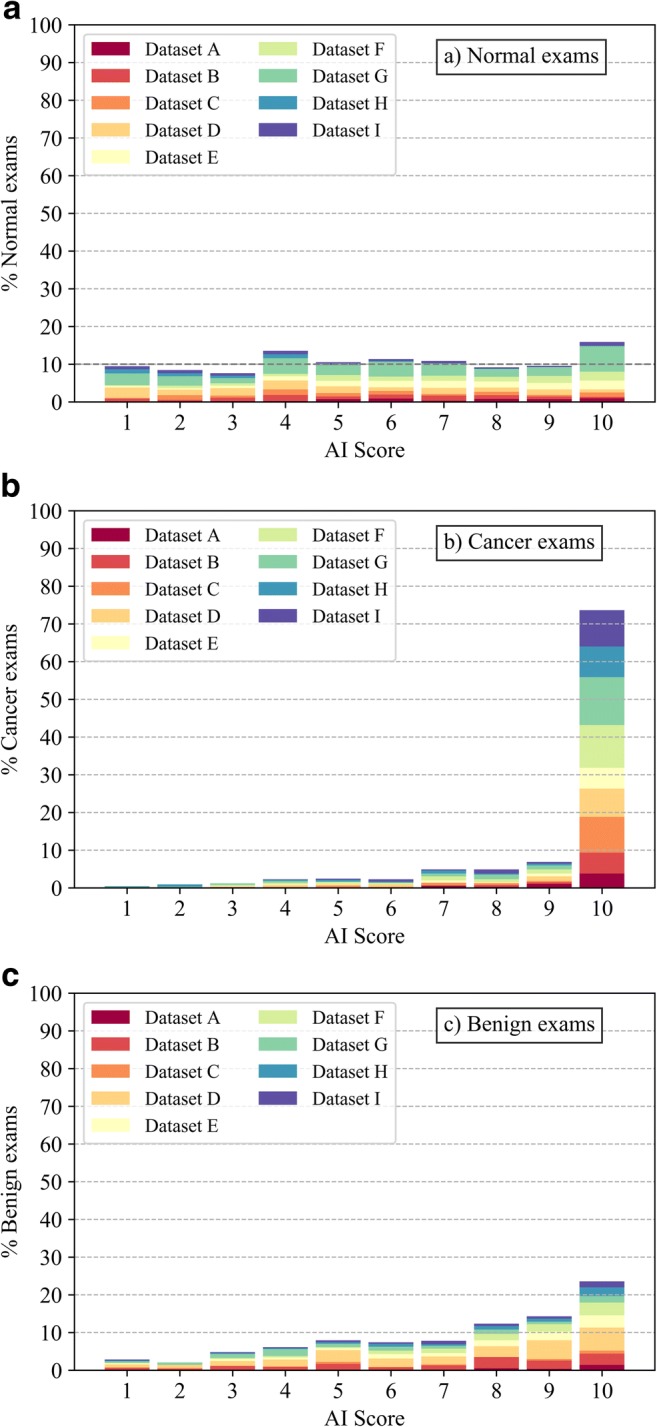
Distribution of normal (**a**), cancer (**b**), and benign exams (**c**) as a function of AI score, representing the likelihood of cancer present (1–10, 10 means high likelihood of cancer present). The contribution of each dataset to the overall percentage of exams is shown

**Fig. 2:**
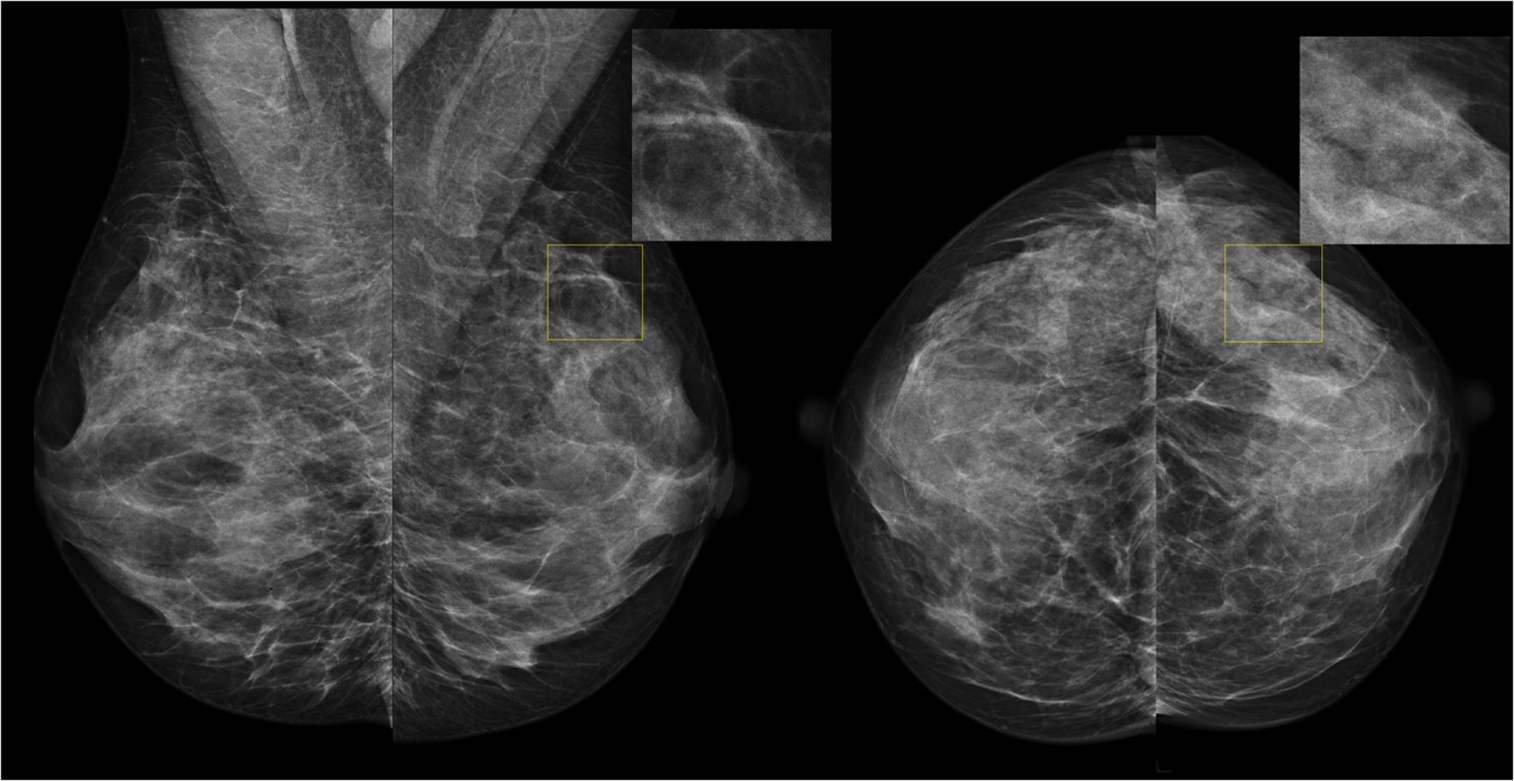
An example of the nine exams in our study that contained cancer but were assigned an AI score of 1 or 2, the lowest cancer-present likelihood categories. None of the 6 radiologists recalled this exam during the original MRMC study (read without priors), suggesting that the cancer visibility with mammography is poor in these exams (and in fact, the cancer may have been detected by other means)

The distributions of DM exams as a function of AI score are shown in Fig. 1 (for each type of exam according to ground truth: a, normal; b, cancer; c, benign). As expected, normal exams are distributed evenly across AI scoring categories, with an average of 10.0% of normal exams per category (range 7.2–14.9%). For the exams containing cancer, 72.5% are categorized within the highest cancer-present likelihood category (10), whereas 95.1% lie in the categories 5–10. In comparison, only 27% of exams containing benign lesions are in category 10. An example of an exam with cancer that was assigned a low AI score is shown in Fig. 2.

### Automated pre-selection of cases

**Fig. 3:**
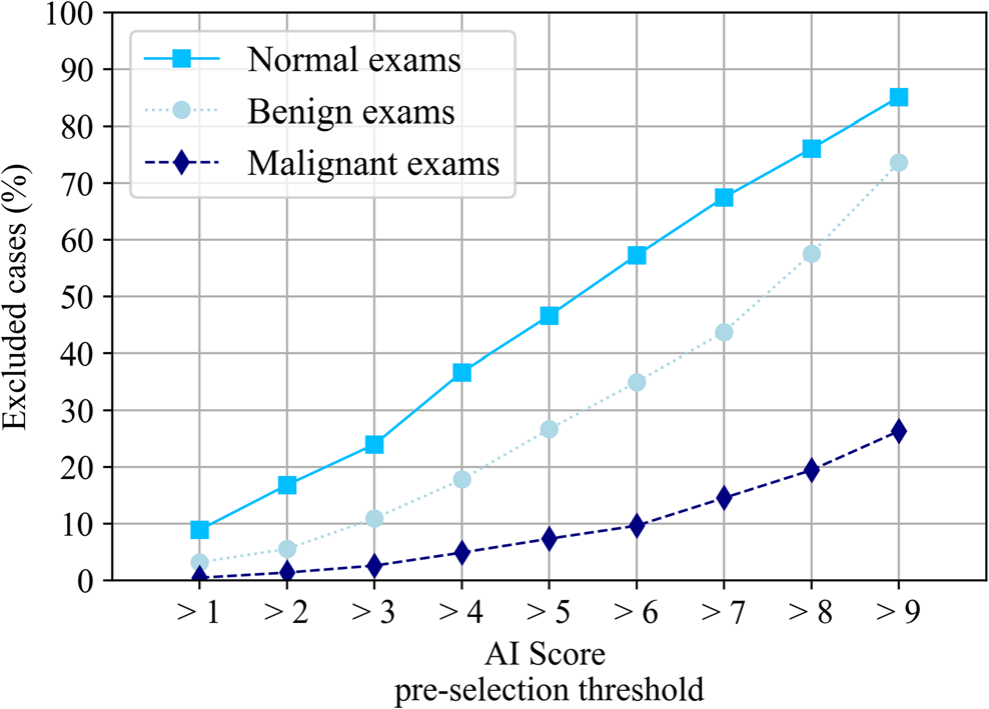
Proportion (%) of exams that would be excluded from the final sample to be evaluated by the radiologists, using all possible AI scores as thresholds values for pre-selection for reading

The proportion and type of exams that would be excluded from reading after a pre-selection of exams using different pre-selection thresholds are depicted in Fig. 3. The trade-off between reducing screening reading workload (e.g., excluding normal exams) and excluding exams containing cancer from the reading is shown: halving the workload of screening (− 47% of screening exams) can be achieved if only exams with scores higher than 5 are read, at the expense of excluding 7% of cancer exams. With a threshold of 2 for pre-selection, for instance, only 1% of exams containing cancer are excluded but the reading workload is reduced by up to 17%. Simultaneously, these thresholds would reduce the cases containing benign lesions by 27% and 5%, respectively, thus reducing the number of false-positive recalls substantially.

**Fig. 4:**
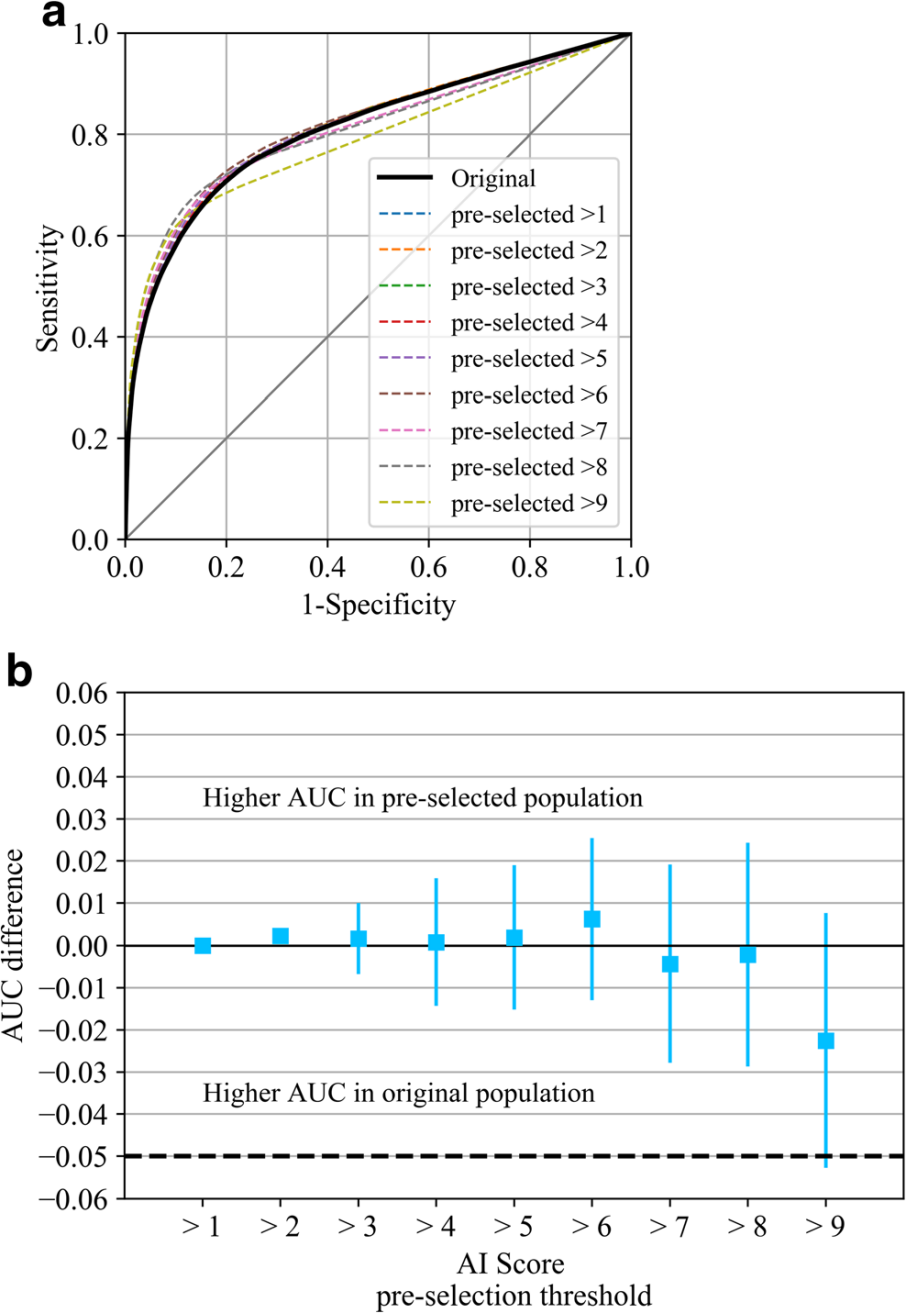
ROC curves (**a**) and change (**b**) in AUC values of the average of radiologists in the original population, as well as in all possible pre-selected populations (using all possible AI scores as threshold values for pre-selection for reading; if the case is not pre-selected, the radiologist score is converted to the lowest possible cancer suspicion score for the MRMC study). 95% confidence intervals are Bonferroni-corrected

After the pre-selection of exams, assuming invariance of reader behavior, the average breast cancer detection performance (AUC) of the radiologists did not change. The AUC for the reading of the pre-selection population was non-inferior to the AUC of the original population. This was significant (lowest Bonferroni-corrected 95% CI > − 0.05, AUC differences were less than 1%, see Fig. 4) for all possible thresholds except for 9, when only exams in the highest cancer-present likelihood category (10) would be evaluated (low 95% CI = − 0.052, AUC decreased by 2%).

## Discussion

In this work, we have evaluated the feasibility of using an AI system to automatically discriminate between screening mammography exams that have a higher and a lower likelihood that cancer is present. Our study shows that, in some situations, it could be a possibility to exclude exams with a lower AI score from human evaluation since the cancer prevalence in these exams is much lower than in an unselected population, thus potentially reducing the reading workload for radiologists.

If such a pre-selection scenario is to be considered, our results point to a trade-off between reducing workload and risking to exclude exams with cancer, which depends on the threshold chosen to create the two groups. On the other hand, the optimal threshold would likely be dependent on local practices and necessities. With a low threshold, we observed a relatively safe scenario with an approximate reduction of 17% in workload at the expense of excluding 1% of exams with cancer from reading (at a cancer detection rate in screening of approximately 6/1000, the cancer prevalence in this group would be approximately 0.3 per 1.000). With a threshold set at an AI score of 5, the workload reduction increases to 47%, at an expense of 7% of cancers. Nevertheless, the exams with cancer in this study do not only originate from screening but also from clinical practice, and it is reported that some cancers in the original reader study were detected by other means such as palpation, ultrasound or breast tomosynthesis [19–26]. This likely means that the reported percentage of excluded exams containing cancer in this study might be overestimating the actual exclusion proportion of screen-detected cancers. A limitation of our study is that we cannot analyze the abovementioned results per detection mode (screening or clinical), per histopathological type of cancers or per breast density, because this information is not available from the original studies.

Our results suggest that pre-selection of exams does not lead to a reduction of the overall detection performance of radiologists, with the AUC varying by less than 1%. This supports the theory that cancers missed by AI are also missed by radiologists, probability due to their low mammographic visibility.

We assumed invariance in reading of the pre-selected mammograms. However, in actual screening practice, there might be several factors affecting radiologists’ scoring of pre-selected exams. The higher prevalence of cancer in the pre-selected cohort might lead to a higher sensitivity for breast cancer, as in practice, it is easier to detect abnormalities when their frequency is relatively high [8]. Likewise, it would be interesting to investigate whether reading the pre-selected group of mammograms in a specific order, e.g., from higher to lower AI score, is also of added value (as cancer prevalence increases with increasing AI score). It would also be possible to increase the recall rate in the pre-selected cohort on purpose (by lowering the recall threshold), in order to counter-balance the exclusion of the cancer-containing mammograms with a low AI score. The fact that more benign lesions are excluded likely makes this possible without increasing the overall recall rate.

The exclusion of cases with benign lesions likely improves the specificity of screening, thus reducing possible harms associated with false-positive recalls. The similarity of ROC curves after exclusion of cases suggest indeed that the negative effect of dismissing exams with cancer is partially balanced by dismissing also false-positive assessments. However, it may be assumed that most benign abnormalities with a relatively low AI score will be lesions that are classified as certainly benign by breast radiologists without biopsy; therefore, the effect on the fraction of women that undergoes biopsy for benign lesions may be smaller.

While in this study we propose automatic labeling of mammograms that would never be read by human radiologists, an alternative possibility is to use the automatically created groups of exams to differentiate cases that need double reading, from cases for which single reading is sufficient. Such a strategy may be valuable for e.g. European screening programs, where double reading is a practice, and may be of special interest for programs that use breast tomosynthesis as the imaging technique for screening, because of the longer reading time per case [25, 35]. Obviously, the effects of such stratification should be further evaluated.

Improvements of the computer system, such as inclusion of temporal information from prior exams, will presumably further enhance the pre-selection, as the current system only uses information from the current DM exams. Evaluation of other systems and versions should be regularly performed considering the rapid speed of evolution in the field of machine learning.

Our study had several limitations. The used datasets were not obtained from screening, but were enriched with cancer cases. The exams were not double-read, as is common practice in screening in Europe, but independently read by multiple radiologists per case. The mix of screening and clinical data may have also introduced cancers that have different characteristics from screen-detected cancers, which might bias our results. Since we have no histological characteristics of the tumors, we cannot be certain of the impact of the cancers that were excluded from human reading based upon the AI score on women’s health. However, because mass screening per se is a balance between benefits, harms, and costs for the society, pre-selection of possibly abnormal and definitively normal cases may be a valid alternative for current screening practice. Further testing of such a pre-selection scenario in real screening populations is required to validate our findings in terms of the effect on recall rates, true-positive and false-positive screening assessments, and interval cancer rates.

In conclusion, we present a new strategy to reduce the reading workload in mammography-based breast cancer screening programs which do not appear to decrease the detection performance of radiologists, by using an AI system to automatically pre-select exams for radiologist evaluation while excluding those exams which have a low likelihood of harboring cancer from human reading.

## Abbreviations

AI: Artificial intelligence
AUC: Area under the curve
BI-RADS: Breast imaging reporting and data system
CAD: Computer-aided detection
CI: Confidence interval
DM: Digital mammography
PoM: Probability of malignancy
ROC: Receiver operating characteristic
